# FREO_2_: An electricity free oxygen concentrator

**DOI:** 10.15172/pneu.2015.6/529

**Published:** 2015-12-01

**Authors:** Bryn A. Sobott, David J. Peake, James F. P. Black, Roger P. Rassool

**Affiliations:** 114 School of Physics, The University of Melbourne, Parkville, Victoria Australia; 214 The Nossal Institute for Global Health, Melbourne School of Population and Global Health, Parkville, Victoria Australia

**Keywords:** oxygen concentrator, sub-district health centre, pneumonia, developing country, children

## Abstract

The World Health Organization recommends oxygen therapy for children with severe pneumonia, but this essential medicine is unavailable in many health centres in limited-resource settings. To address this need, an appropriate means of oxygen provision will need to be low-cost and robust, require little maintenance and not compete for fuel with other vital functions, and be environmentally sustainable. This report presents the preliminary results of the Fully Renewable Energy Oxygen (FREO2) system, confirming the viability of a novel means of producing medical grade oxygen without any electricity. The approach relies on exploiting the reduction in pressure of water flowing through a raised siphon to create a source of vacuum. This is used to power a customised vacuum-pressure-swing-adsorption system and produce medical grade oxygen. The FREO2 system has been designed to meet the criteria for successful oxygen delivery in small health facilities. It is ideally suited for deployment in tropical or mountainous regions with proximity to flowing water. Importantly, the oxygen generating capacity of FREO2 rises with the increased demand commonly observed during the rainy season in such climates.

## 1. Introduction

### 1.1 The problem

Oxygen therapy is recommended by the World Health Organization as an essential medicine for the treatment of children with severe pneumonia [1]. However, in limited-resource settings the high cost of bottled oxygen and logistical difficulties in its distribution mean that oxygen is not readily available in many (if not most) district and sub-district health facilities. It has been estimated that 122,000 children’s lives could be saved annually by improved provision of oxygen globally [2]. In Papua New Guinea bottled oxygen costs about 10 times as much (averaged over two years) as delivery via concentrators [3].

Addressing the lack of therapeutic oxygen in such health facilities in developing countries presents a range of challenges which have to be overcome. Any viable solution will need to satisfy a number of challenging criteria. It must have minimal capital cost and work under harsh conditions, including high temperature and humidity, and in dusty environments. It should be robust and reliable, require little maintenance, and be simple enough to be repaired by a local mechanic. Competition for fuel with other vital functions of the health facility, such as lighting or transport, should be avoided. Most importantly, its energy source should be low-cost, continuous and environmentally sustainable.

Conventional oxygen concentrators offer a potential solution. These typically provide sufficient oxygen for one adult patient at a time, or up to four paediatric patients [4]. These devices contain ‘molecular sieves’ of zeolites, which are made from an abundantly available, low-cost mineral. When air is compressed and brought into contact with zeolite, nitrogen in the air is preferentially adsorbed over oxygen. As the pressure is reduced, the trapped nitrogen can be released again. A cyclic process, known as pressure-swing adsorption [5] can be used to remove nitrogen from the air in this way. Concentrators typically combine two canisters of zeolite with an air compressor, tapping the oxygen from the canister under compression while venting the nitrogen from the other. This cycling of compression and decompression creates a continuous flow of up to 95% pure oxygen. There is growing evidence that standard concentrators can be a viable source of oxygen in some developing country health facilities [6,7].

However, currently available oxygen concentrators use an electrical air compressor to provide the required pressure swing. As many small health facilities in low- and middle-income countries do not have reliable, 24-hour access to electrical power supply [8], conventional oxygen concentrators may not be appropriate for these facilities. In a 2006 study in Papua New Guinea, oxygen was not available overall for 13% of children with hypoxaemia presenting to hospitals, with remote rural district hospitals having the lowest rates of oxygen availability [9].

This report presents an innovative method to power an oxygen concentrator that uses the renewable energy of a flowing stream or river and has the potential to meet the criteria listed above. Further, seasonal variation in water-flow will allow oxygen-generating capacity to rise with the increased demand commonly observed during the rainy season [10].

## 2. A possible solution

The Fully Renewable Energy Oxygen (FREO_2_) system has three major components: (i) a small amount of water from a flowing stream is directed through a siphon to produce a vacuum; (ii) part of this vacuum is mechanically converted to compressed air; (iii) the remaining vacuum and the compressed air are used to power a unique vacuum pressure swing adsorption system and concentrate oxygen for delivery to the health facility.

### 2.1 Details of the three components

#### 2.1.1 Creating a vacuum using a flowing stream

**Figure 1:**
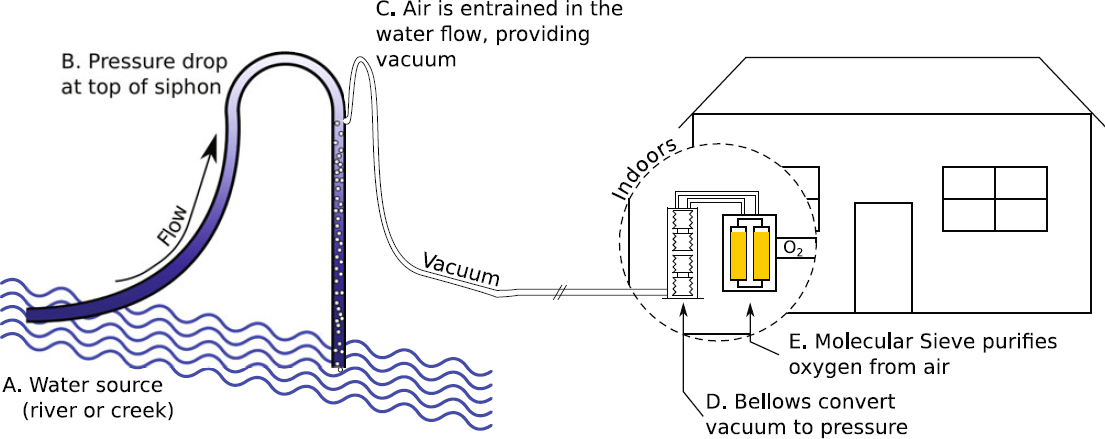
The Fully Renewable Energy Oxygen (FREO_2_) system. Water flowing through a siphon draws in air. This vacuum travels through a pipe to a clinic where a portion is used to compress air. Vacuum and compressed air are alternately applied to a molecular sieve to concentrate oxygen from room air

Water is diverted from a perennial stream in such a way to provide at least 1.5 metres of available height difference (‘head’) between inflow and outflow [11]. As shown in Figure 1, this head can be obtained by running a pipe along an inclined stream. A section of the pipe is raised approximately 7 metres vertically and then directed back into the stream. Towards the top of the pipe (siphon), there is a small orifice through which air can be allowed to enter. In this region, the pressure of the flowing water is significantly below atmospheric pressure. As air is entrained into the stream of water flowing through the siphon, it acts like a pressure interchanger between the two fluids, and a vacuum is produced at this orifice. This vacuum provides the pressure differential required by the oxygen concentration process. The absence of moving parts in the water minimises capital and maintenance costs and there are reports of a similar pressure interchanger operating continuously and maintenance free for more than 50 years [12]. No water is consumed, as the air-carrying water flows back into the same stream. Further, because airflow is in the direction of the stream and this section has no contact with the patients, medical grade materials are not required and the process is independent of water quality.

#### 2.1.2 Using a vacuum to produce compressed air

Converting the vacuum to positive pressure is achieved by mechanically coupling two bellows in a pull—push arrangement. The vacuum created from the siphon is used to draw air out of one of the bellows whilst a second bellow coupled to the first is drawn in, pushed closed. This process is used repeatedly to mechanically convert the vacuum into a pressure. By linking two pairs of bellows, a continuous flow of compressed air can be created (as one pair is compressing air into a zeolite canister, the other is returning to its initial state ready to start the cycle again).

#### 2.1.3 The production of oxygen with FREO_2_

The FREO_2_ system is based on a vacuum-pressure-swing-adsorption cycle. A novel approach is used to repeatedly switch vacuum and pressure between two zeolite canisters without the need of any electrical components. The remaining vacuum from the siphon is used to evacuate the nitrogen from the zeolite canisters once the oxygen has been harvested. The benefits of desorbing the nitrogen at sub-atmospheric pressures are well documented and include reduced energy requirements and reduced susceptibility to high humidity environments [13].

An analysis of the maintenance requirements of oxygen concentrators in low-resource settings has identified sieve bed replacement as the single most expensive repair [14]. To maximise the lifetime of the sieve beds in FREO_2_, the pressure swing cycle has been designed to limit the progression of moisture, and external cleanable filters are used to remove dust.

There are reports of pressure differentials being transported over 6 kilometres with a marginal pressure loss [15]. This indicates that it should be possible to separate the three components of the system and deliver the vacuum several kilometers from the stream. The eventual limiting factor is likely to be economic and related to the cost of the piping.

## 3. Current status and next steps

**Figure 2:**
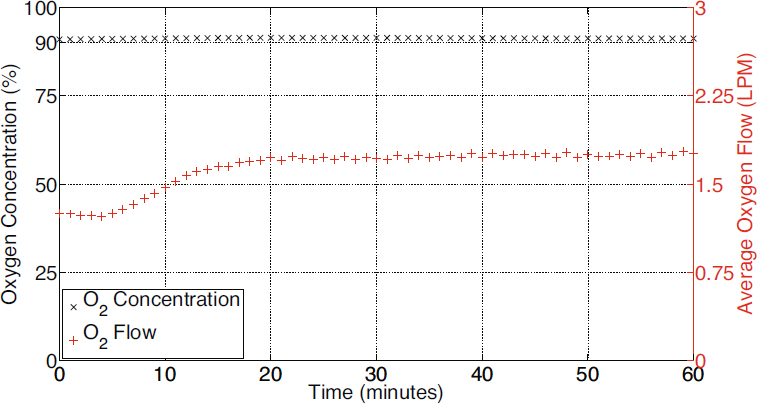
Preliminary results demonstrating the concentration of oxygen to 91±2% using the prototype Fully Renewable Energy Oxygen (FREO_2_) system

A fully functional FREO_2_ prototype has been constructed and preliminary testing of the system has been undertaken on a small creek in rural Australia. A data acquisition system was created in-house to monitor and record the performance of the system. Oxygen purity and flow rate were continuously monitored with a Servomex 1440 gas analyser (Earnie Graves Company, USA) and a Sensirion SFM3000 mass flow meter (Sensirion AG, Switzerland), respectively. Preliminary results showing 91±2% (systematic error) oxygen produced at a mean flow rate of 1.7±0.1 LPM (litres per minute) are presented in Figure 2.

At regular intervals, the water flow through the siphon was monitored using an Omni TDI-200H ultrasonic flow meter (Omni Instruments, UK). The results presented in Figure 2 were achieved with a head of approximately 4 metres and 540 LPM of water flow. These conditions were imposed by the test site and are not ideal as earlier work indicates that maximum efficiency can be achieved by reducing the head to less than 1.7 metres [11]. Consequently it is anticipated that oxygen can be produced more efficiently by reducing the head. Further, the approach is scalable. If more water is available, oxygen flow rate can be increased. An international patent application based on this method has been filed [16].

Optimisation of each component is now underway with the goal of preparing the prototype for field trialling in a developing country health facility. A range of field trials is anticipated within six months, including studies of the reliability of the bellows and sieve beds. We will also investigate the actual requirements for oxygen in a typical small health facility. There is little empirical data to advise how often such a clinic would need more than this amount. A feasibility study in Papua New Guinea [3] suggests that a district hospital would require 17,000 l/day. A single FREO_2_ unit will be capable of supplying about 6,000 l/day, most likely sufficient for a sub-district health centre. We will also collaborate with health workers to assess training requirements and the need for consumables such as oxygen tubing and nasal prongs.

## 4. Conclusion

The FREO_2_ system offers a sustainable and workable solution for health facilities in remote areas with limited access to oxygen cylinders or a reliable electricity supply. To the best of our knowledge, the concentration of oxygen without electricity has not been previously described. In the interest of cost, maintenance, robustness and efficiency, unnecessary energy conversions are avoided. Further, by not competing with other uses of fuels such as diesel and electricity, FREO_2_ is designed to promote health equity in sub-district health facilities. Water is not consumed, so the approach is fully renewable and multiple siphons could be multiplexed to meet demand.

Significant progress is still required, and this solution is not appropriate everywhere that oxygen is needed. However, for health facilities within range of an appropriate water source, FREO_2_ has the potential to be a life-saving new technology that could impact on pneumonia child mortality.
